# Complete genome sequence of *Staphylothermus marinus* Stetter and Fiala 1986 type strain F1

**DOI:** 10.4056/sigs.30527

**Published:** 2009-09-25

**Authors:** Iain J. Anderson, Hui Sun, Alla Lapidus, Alex Copeland, Tijana Glavina Del Rio, Hope Tice, Eileen Dalin, Susan Lucas, Kerrie Barry, Miriam Land, Paul Richardson, Harald Huber, Nikos C. Kyrpides

**Affiliations:** 1 Joint Genome Institute, 2800 Mitchell Drive, Walnut Creek, California, USA; 2 Bioscience Division, Oak Ridge National Laboratory, Oak Ridge, Tennessee, USA; 3 Lehrstuhl für Mikrobiologie und Archaeenzentrum, Universität Regensburg, Regensburg, Germany

**Keywords:** *Archaea*, *Desulfurococcales*, sulfur-reducing, hyperthermophile

## Abstract

*Staphylothermus marinus* Fiala and Stetter 1986 belongs to the order *Desulfurococcales* within the archaeal phylum *Crenarchaeota*. *S. marinus* is a hyperthermophilic, sulfur-dependent, anaerobic heterotroph. Strain F1 was isolated from geothermally heated sediments at Vulcano, Italy, but *S. marinus* has also been isolated from a hydrothermal vent on the East Pacific Rise. We report the complete genome of *S. marinus* strain F1, the type strain of the species. This is the fifth reported complete genome sequence from the order *Desulfurococcales*.

## Introduction

**Figure 1:**
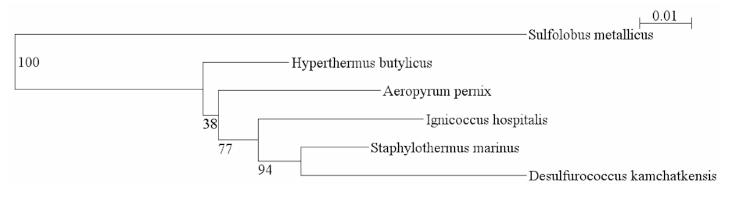
Phylogenetic tree of 16S ribosomal RNA of members of the order *Desulfurococcales* with completely sequenced genomes. *Sulfolobus metallicus* is the outgroup. The tree was generated with weighbor through the Ribosomal Database Project [3] and viewed with njplot [4].

Strain F1 is the type strain of the species *Staphylothermus marinus*. It was isolated from geothermally heated sediments at Vulcano, Italy [1], and was the strain sequenced. *S. marinus* was also isolated from a hydrothermal vent on the East Pacific Rise. There is one other species within the genus, *Staphylothermus hellenicus*, which was isolated from a hydrothermal vent at Milos, Greece [2]. Four other complete genomes from the order *Desulfurococcales* have been published, but *S. marinus* is not closely related to any of these organisms (Figure 1).

*S. marinus* is a nonmotile coccus with a diameter of 0.5-1.0 μm. At low nutrient concentrations it forms clumps of up to 100 cells, while at higher nutrient concentrations single cells or pairs of cells are observed. At high concentrations of yeast extract, giant cells with a diameter of up to 15μm are formed [1]. The optimum and maximum growth temperatures also depend on the nutrient concentration. At low nutrient concentration the optimum growth temperature is 85°C and the maximum is 92°C, while at higher nutrient concentration the optimum growth temperature is 92°C and the maximum is 98°C [1]. The optimum pH for growth is 6.5, but growth is observed within a range of 4.5 to 8.5.

*S. marinus* is a heterotroph, growing on complex media but not on simple carbohydrates or amino acids. Elemental sulfur is required for growth, and it can not be substituted by other sulfur compounds [1]. In the absence of sulfur, cells can survive while producing hydrogen [5]. Metabolic products are CO_2_, H_2_S, acetate, and isovalerate, suggesting a metabolism similar to that of *Pyrococcus* species [1].

We describe here the properties of the complete genome sequence of *S. marinus* strain F1 (DSM 3639, ATCC 43588).

### Classification and features

Several features suggest that *S. marinus* is a typical member of the *Archaea*. Its growth was not inhibited by vancomycin, kanamycin, streptomycin, or chloramphenicol, but it is sensitive to diphtheria toxin [1]. Its cell wall lacks murein, and it contains typical archaeal membrane lipids [1]. Other features of the organism are presented in Table 1.

**Table 1:** Classification and general features of *S. marinus* F1 according to the MIGS recommendations [6].

MIGS ID	Property	Term	Evidence code
		Domain *Archaea*	TAS [7]
		Phylum *Crenarchaeota*	TAS [8,9]
		Class *Thermoprotei*	TAS [9,10]
	Current classification	Order *Desulfurococcales*	TAS [11,12]
		Family *Desulfurococcaceae*	TAS [13-15]
		Genus *Staphylothermus*	TAS [1]
		Species *Staphylothermus marinus*	TAS [1]
	Gram stain	negative	TAS [1]
	Cell shape	coccus	TAS [1]
	Motility	nonmotile	TAS [1]
	Sporulation	nonsporulating	NAS
	Temperature range	65-98°C	TAS [1]
	Optimum temperature	85-92°C	TAS [1]
MIGS-6.3	Salinity	1-3.5% NaCl	TAS [1]
MIGS-22	Oxygen requirement	anaerobe	TAS [1]
	Carbon source	peptides	TAS [1]
	Energy source	peptides	TAS [1]
MIGS-6	Habitat	marine geothemally heated areas	TAS [1]
MIGS-15	Biotic relationship	free-living	TAS [1]
MIGS-14	Pathogenicity	none	NAS
	Biosafety level	1	NAS
	Isolation	geothermally heated sediment	TAS [1]
MIGS-4	Geographic location	Vulcano, Italy	TAS [1]
MIGS-5	Isolation time	1984	TAS [1]
MIGS-4.1 MIGS-4.2	Latitude-longitude	38.4/15.0	TAS [1]
MIGS-4.3	Depth	0.5 m	TAS [1]
MIGS-4.4	Altitude	not applicable	

Evidence codes - IDA: Inferred from Direct Assay (first time in publication); TAS: Traceable Author Statement (i.e., a direct report exists in the literature); NAS: Non-traceable Author Statement (i.e., not directly observed for the living, isolated sample, but based on a generally accepted property for the species, or anecdotal evidence). These evidence codes are from the Gene Ontology project [16]. If the evidence code is IDA, then the property was observed for a living isolate by one of the authors or an expert mentioned in the acknowledgements.

## Genome sequencing and annotation

### Genome project history

*S. marinus* was selected for sequencing based upon its phylogenetic position relative to other sequenced archaeal genomes. It is part of a 2006 Joint Genome Institute Community Sequencing Program (CSP) project that included six diverse archaeal genomes. The complete genome sequence was finished in February, 2007. The GenBank accession number for the chromosome is CP000575. The genome project is listed in the Genomes OnLine Database (GOLD) [17] as project Gc00511. Sequencing, finishing and annotation were performed by the DOE Joint Genome Institute (JGI). A summary of the project information is shown in Table 2.

**Table 2:** Genome sequencing project information.

**MIGS ID**	**Property**	**Term**
MIGS-28	Libraries used	3kb, 6kb and 40kb (fosmid)
MIGS-29	Sequencing platform	ABI3730
MIGS-31.2	Sequencing coverage	13.3×
MIGS-31	Finishing quality	Finished
	Sequencing quality	less than one error per 50kb
MIGS-30	Assembler	Phrap
MIGS-32	Gene calling method	CRITICA, Glimmer
	GenBank ID	CP000575
	GenBank date of release	February 2007
	GOLD ID	Gc00511
	NCBI project ID	17449
	IMG Taxon ID	640069332
	Project relevance	Tree of Life

### DNA isolation, genome sequencing and assembly

The methods for DNA isolation, genome sequencing and assembly for this genome have previously been published [18].

### Genome annotation

Protein-coding genes were identified using a combination of Critica [19] and Glimmer [20] followed by a round of manual curation using the JGI GenePRIMP pipeline [21]. The predicted CDSs were translated and used to search the National Center for Biotechnology Information (NCBI) nonredundant database, UniProt, TIGRFam, Pfam, PRIAM, KEGG, COG, and InterPro databases. The tRNAScan-SE tool [22] was used to find tRNA genes. Additional gene prediction analysis and manual functional annotation was performed within the Integrated Microbial Genomes Expert Review (IMG-ER) platform [23].

### Genome properties

**Figure 2:**
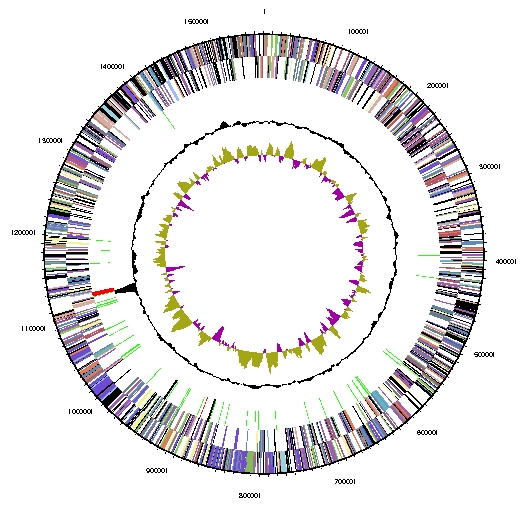
Graphical circular map of the chromosome. From outside to the center: Genes on forward strand (colored by COG categories), Genes on reverse strand (colored by COG categories), RNA genes (tRNAs green, rRNAs red, other RNAs black), GC content, GC skew.

The genome of *S. marinus* F1 consists of a single circular chromosome (Table 3 and Figure 2). The genome size of 1.57 Mbp is smaller than most *Crenarchaeota*, although *Desulfurococcus kamchatkensis* and *Ignicoccus hospitalis* have smaller genomes. The G+C percentage is 35.7%, lower than that of most *Crenarchaeota*. Among *Crenarchaeota* with sequenced genomes, only *Sulfolobus tokodaii* has a lower G+C percentage (32.8%). The total number of genes is 1,659, with 1,610 protein-coding genes and 49 RNA genes. There are 40 pseudogenes, constituting 2.4% of the total genes. The percentage of the genome encoding genes (89.1%) is close to the average for *Crenarchaeota*. About 59% of predicted genes begin with an AUG codon, 33% begin with UUG, and only 8% begin with GUG. There is one copy of each ribosomal RNA. The properties and statistics of the genome are shown in Table 3, and the distribution of proteins in COG categories is shown in Table 4.

**Table 3:** Genome statistics

**Attribute**	**Value**	**% of total**
Genome size (bp)	1,570,485	100.00%
DNA coding region (bp)	1,399,620	89.1%
DNA G+C content (bp)	561,080	35.7%
Number of replicons	1	
Extrachromosomal elements	0	
Total genes	1659	100.00%
RNA genes	49	3.0%
rRNA operons	1	
Protein-coding genes	1610	97.0%
Pseudogenes	40	2.4%
Genes with function prediction	974	60.5%
Genes in paralog clusters	542	33.7%
Genes assigned to COGs	1109	68.9%
Genes assigned Pfam domains	1089	67.6%
Genes with signal peptides	317	19.7%
Genes with transmembrane helices	348	21.6%
CRISPR repeats	12	

**Table 4:** Numbers of genes associated with the 25 general COG functional categories.

**Code**	**value**	**%**	**Description**
E	74	4.6	Amino acid transport and metabolism
G	72	4.5	Carbohydrate transport and metabolism
D	8	0.5	Cell cycle control, cell division, chromosome partitioning
N	4	0.2	Cell motility
M	23	1.4	Cell wall/membrane/envelope biogenesis
B	2	0.1	Chromatin structure and dynamics
H	53	3.3	Coenzyme transport and metabolism
Z	0	0.0	Cytoskeleton
V	17	1.1	Defense mechanisms
C	92	5.7	Energy production and conversion
W	0	0.0	Extracellular structures
S	116	7.2	Function unknown
R	199	12.4	General function prediction only
P	85	5.3	Inorganic ion transport and metabolism
U	12	0.7	Intracellular trafficking, secretion, and vesicular transport
I	15	0.9	Lipid transport and metabolism
Y	0	0.0	Nuclear structure
F	39	2.4	Nucleotide transport and metabolism
O	53	3.3	Posttranslational modification, protein turnover, chaperones
A	2	0.1	RNA processing and modification
L	71	4.4	Replication, recombination and repair
Q	5	0.3	Secondary metabolites biosynthesis, transport and catabolism
T	18	1.1	Signal transduction mechanisms
K	60	3.7	Transcription
J	164	10.2	Translation, ribosomal structure and biogenesis
-	426	26.5	Not in COGs

## Insights from genome sequence

The genome of *S. marinus* has several novel features compared to other *Crenarchaeota*. It is the first crenarchaeote found to have a sodium ion-translocating decarboxylase, which is probably involved in energy generation from amino acid degradation [18]. In addition it is the first crenarchaeote found to have proteins related to multisubunit cation/proton antiporters, although the *S. marinus* proteins probably do not function as antiporters. These antiporter-related proteins belong to larger operons similar to the *mbh* and *mbx* operons of *Pyrococcus furiosus* [24,25], therefore, they may play a role in sulfur reduction or hydrogen production. *S. marinus* appears to use different proteins for sulfur reduction than the other anaerobic, sulfur-reducing *Crenarchaeota*. Both *Thermofilum pendens* and *Hyperthermus butylicus* appear to have molybdenum-containing sulfur/polysulfide reductases and NADPH:sulfur oxidoreductases, but these are not present in *S. marinus* [18]
